# Ultrasound for Distal Forearm Fracture: A Systematic Review and Diagnostic Meta-Analysis

**DOI:** 10.1371/journal.pone.0155659

**Published:** 2016-05-19

**Authors:** Djoke Douma-den Hamer, Marco H. Blanker, Mireille A. Edens, Lonneke N. Buijteweg, Martijn F. Boomsma, Sven H. van Helden, Gert-Jan Mauritz

**Affiliations:** 1 Department of Emergency Medicine, Isala hospital, Zwolle, the Netherlands; 2 Department of General Practice, University Medical Center Groningen, University of Groningen, Groningen, the Netherlands; 3 Department of Clinical Epidemiology, Isala hospital, Zwolle, the Netherlands; 4 Department of Radiology, Isala hospital, Zwolle, the Netherlands; 5 Department of Trauma Surgery, Isala hospital, Zwolle, the Netherlands; Klinikum rechts der Isar - Technical University Munich - TUM, GERMANY

## Abstract

**Study Objective:**

To determine the diagnostic accuracy of ultrasound for detecting distal forearm fractures.

**Methods:**

A systematic review and diagnostic meta-analysis was performed according to the PRISMA statement. We searched MEDLINE, Web of Science and the Cochrane Library from inception to September 2015. All prospective studies of the diagnostic accuracy of ultrasound versus radiography as the reference standard were included. We excluded studies with a retrospective design and those with evidence of verification bias. We assessed the methodological quality of the included studies with the QUADAS-2 tool. We performed a meta-analysis of studies evaluating ultrasound to calculate the pooled sensitivity and specificity with 95% confidence intervals (CI95%) using a bivariate model with random effects. Subgroup and sensitivity analysis were used to examine the effect of methodological differences and other study characteristics.

**Results:**

Out of 867 publications we included 16 studies with 1,204 patients and 641 fractures. The pooled test characteristics for ultrasound were: sensitivity 97% (CI95% 93–99%), specificity 95% (CI95% 89–98%), positive likelihood ratio (LR) 20.0 (8.5–47.2) and negative LR 0.03 (0.01–0.08). The corresponding pooled diagnostic odds ratio (DOR) was 667 (142–3,133). Apparent differences were shown for method of viewing, with the 6-view method showing higher specificity, positive LR, and DOR, compared to the 4-view method.

**Conclusion:**

The present meta-analysis showed that ultrasound has a high accuracy for the diagnosis of distal forearm fractures in children when used by proper viewing method. Based on this, ultrasound should be considered a reliable alternative, which has the advantages of being radiation free.

## Introduction

Distal forearm fractures are common both in adults and the paediatric age group. In the latter group, these fractures are mostly localized extra-articular [1,2], in contrast to adults who are more likely to have an intra-articular component [3,4]. Conventional x-rays of the forearm in two planes are considered the gold standard for fracture detection and to guide treatment. The last two decades ultrasound has emerged as a possible alternative for fracture identification in the Emergency Department (ED). One of the main advantages is the absence of exposure to ionizing radiation. Because children are up to four times more radiation sensitive and have a higher risk of being exposed to cumulative doses of radiation over time [5–9], ultrasound has received greater interest in this age group. Other advantages of ultrasound may be the relative easiness to teach [10–13], reduced pain experience [14,15], and the provision of additional information about the musculoskeletal system [16–18]. Whether ultrasound can be used as a primary screening tool is currently under debate [15,19–22]. It has been suggested that ultrasound only provides additional value under special circumstances, like the pre hospital environment, disaster areas, developing countries, suspicion of occult fracture in poorly ossified bones, pregnant patients and to reduce exposure to serial direct radiographs in fracture reduction [23–27]. An important feature in this debate is the actual diagnostic accuracy of ultrasound for detecting forearm fractures.

In this systematic review and diagnostic meta-analysis, we aim to assess the diagnostic accuracy of ultrasound for distal forearm fractures using radiography as a reference standard. The secondary goal was to investigate the effect of training, methods of scanning, bones scanned (ulna, radius or combined), probe frequency, age, fracture, and reposition rate.

## Methods

We conducted a systematic review, using the Preferred Reporting Items for Systematic Reviews and Meta-Analyses (PRISMA) checklist.

### Data sources and searches

We systematically searched the literature using Pubmed (1946-7^th^ September 2015), Embase (1974-7^th^ September 2015) and the Cochrane Database of Systematic Reviews. We combined MeSH headings or Emtrees and key words to identify eligible studies (for details see S1 Text). There were no restrictions incorporated in the search itself. The search was supplemented by manually reviewing the reference lists of all the retrieved articles.

### Study selection

We imported all the references into a bibliographic database (www.refworks.com) and removed duplicates. Two reviewers (DD, GJM) independently reviewed titles and abstracts for possible inclusion. Candidate publications were read full text, and included if original research was reported on the comparison of ultrasound and conventional x-ray in patients with possible distal forearm fractures, and the publication was written in English, French, German, or Spanish. We excluded case reports, editorials, letters to editors, and conference proceedings only.

### Data extraction and quality assessment

From each included publication two reviewers extracted the following data: sample size, sampling method (consecutive, convenience), year of publication, land of conducting the study, demographic characteristics of the patients including age, gender, the number of patients with/without distal radius fracture according to the results of conventional x-ray, reposition rate (percentage of patients with fracture undergoing reposition), characteristics of ultrasound device (transducer, probe frequency), its operator (trained, untrained), method of visualization (4 versus 6-view), outcomes recorded for radius and ulna separate, distal forearm seen as one entity and distal forearm seen as two separate bones.

We assessed the methodological quality of each study, using the Quality Assessment of Diagnostic Accuracy Studies (QUADAS-2) criteria, which provides a standardized approach to grade the quality of studies included in a meta-analysis [28]. The tool is composed of two parts: the risk of bias (four domains: patient selection, index test, reference standard, and flow and timing) and concerns regarding applicability (three domains: patient selection, index test, and reference standard). All items are categorized as low, unclear or high risk of bias. Both reviewers scored the 7-item tool independently and disagreements were solved by discussion.

### Statistical analysis

The accuracy of ultrasound for the detection of fractures is assessed with conventional x-ray as reference standard comparator. The two reviewers (DD, GJM) independently extracted data in absolute numbers of true positive (TP), false positive (FP), false negative (FN), and true negative (TN) from the included articles. Web based programs were used to calculate these numbers from the article if only sensitivity and specificity were presented. When these data could not be obtained from the article by calculation, the articles were excluded from the study.

For the main comparison, coupled forest plots are presented, depicting sensitivity and specificity, together with a hierarchical summary receiver operator curve (HSROC). Separate pooling of sensitivity and specificity estimates will not be presented, as these fail to account for the trade-off between sensitivity and specificity, which may lead to underestimates of test accuracy [29]. The pooled estimates, were, however, used to derive the pooled diagnostic odds ratio (DOR).

Heterogeneity is to be expected in meta-analyses of diagnostic accuracy. Therefore, random effects models are fitted. This will provide an estimate of the average accuracy of the test and describe the variability in this effect.

The traditional I^2^ statistics is not recommended for quantifying heterogeneity in sensitivity and specificity because it is a univariate measure that does not account for potential threshold effects [30]. To investigate whether a factor was associated with test accuracy, we have performed exploratory analyses by visual inspection of the forest plots and HSROC plots. Pooled estimates of sensitivity, specificity, positive and negative likelihood ratio (LR), and DOR are presented with 95% confidence intervals (CI95%) for subgroups of five or more studies.

The following subgroup analyses were planned: bones of the forearm scanned (radius and ulna seen as separate bones or combined as one entity), skills level (trained versus untrained), method of scanning (4-view versus 6-view), probe frequency (< = 7.5MHz or >7.5MHz), radius and ulna separately, age (below and above 18 years), fracture rate (below and above 50%) and reposition rate (below and above 10%).

Two post-hoc analyses were added during the analysis. First, the main analysis was performed on the 16 studies with trained versus untrained data from the study of Chaar-Alvarez *et al*. [14]. Secondly, the main analysis en sub analysis radius/ulna was performed with and without the three studies that only provided information for the radius and not the forearm as one entity [31–33].

All analyses were performed using Stata 14.0 (StataCorp LP, Texas, USA).

## Results

### Overview of literature search

**Fig 1:**
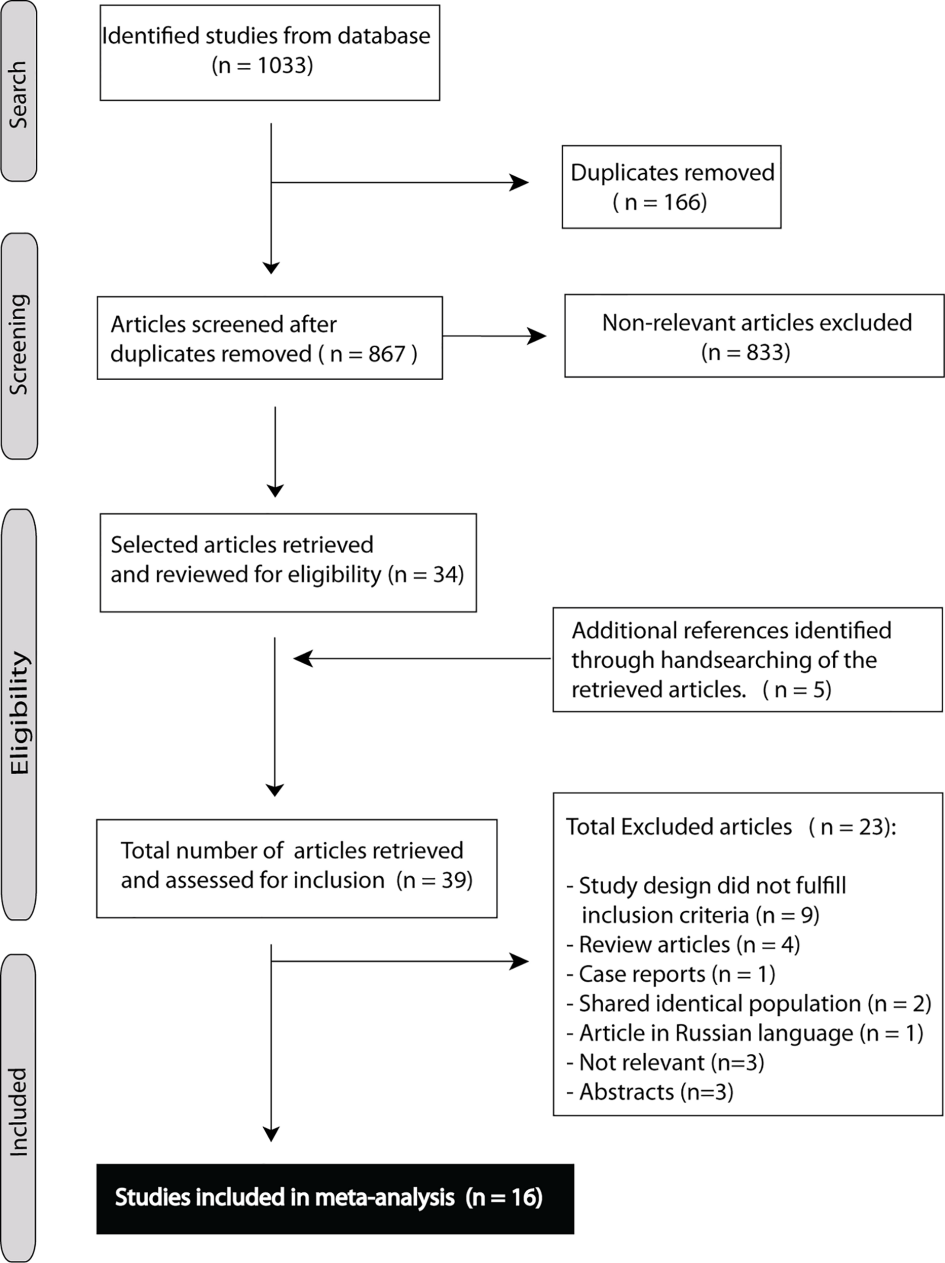
Flowchart of articles retrieved from search of databases and reasons of exclusion.

Out of 867 publication, we included 16 studies (Fig 1) [2,14,20,31–43]. Thirteen studies had data available for TP, FP, FN, TN for the distal forearm seen as one entity [2,14,20,31–43]. Three other studies had unequal data available for the distal radius and ulna separately [31–33], preventing analyses of the distal forearm as one entity. Data from the distal radius were used as a substitute in these three studies, because in case of a fracture of the distal forearm it is mostly the distal radius with or without the ulna and rarely an isolated ulna fracture [35,42,44].

### Characteristics of selected studies

A total of 1,204 patients with 641 fractures were included in the 16 studies. The studies were performed between 2000 and 2015 at different countries worldwide. All studies are prospective studies. The age of subjects ranged from 0 to 88 years old, with most studies (n = 11) focusing on the paediatric up to young adult age groups [2,14,20,31,32,34–38,42]. Male patients compromised 60.4% of the study population. Overall fracture rate was 53.2% (range 25–79.2%) and reposition rate was 27.6% (range 0–76,5%). The characteristics of the individual studies are summarized in Table 1 and Table 2. Only two studies included inter-rater reliability [14,33].

**Table 1:** Characteristics of studies and patients enrolled from studies reviewed for meta-analyses.

Study	Year	Origin	Design	Sample size, n	Forearm, n	Fracture,n	Age (mean), years	Male/Female, n	Reposition, n
**Williamson**	2000	England	Pilot	26	26	16	8	16/10	0
**Hübner**	2000	Germany	Prospective	163	85/54^#^	59/23	NR	NR	NR
**Chen**	2007	USA	Prospective	68	68	48	10	41/27	26
**Mortiz**	2008	Germany	Prospective	653	145	63	4.4	NR	0/NR
**Patel**	2009	USA	Prospective	33	28	19	9.1	22/11	7^^^
**Ackermann**	2010	Germany	Prospective cohort multicenter	93	93	64	8.1	44/49	0
**Weinberg**	2010	USA	Prospective cohort multicenter	212	40/30^#^	14/8	(13)^$^	NR	NR
**Chaar-Alvarez**	2011	USA	Prospective tertiary	101	101	46	10.3 (11)^$^	65/43	3
**Sinha**	2011	India	Prospective Observational	41	16	4	12.7	NR	NR
**Beltrame**	2012	Italy	Prospective	86	12	8	(53)^$^	NR	NR
**Eckert**	2012	Germany	Prospective	115	115	62	9.1	64/51	5
**Waterbrook**	2013	USA	Prospective observational	106	33/26^#^	14/3	34	51/52	NR
**Javadzadeh**	2014	Iran	Prospective	260	134	64	42.63	148/112	NR
**Kozaci**	2015	Turkey	Prospective observational	83	83	55	13.4	65/18	31
**Herren**	2015	Germany	Prospective multicenter	201	201	98	9.5	132/69	34
**Musa**	2015	England	Prospective	97	24	19	NR	NR	NR

^#^ radius and ulna separately, first number is radius, second number is ulna (all the other studies are distal forearm seen as one entity)

^^^ Estimate, 7–13: total of 13 separate bones met criteria for reduction, exact number of combined cannot be derived from the article

^$^ Age with () are median, instead of average

* Chaar-Alvarez trained

^&^ Chaar-Alvarez untrained

NR: not reported.

**Table 2:** Continuation of characteristics of included studies.

	Training^^^	Method of viewing ^/^	Probe frequency, MHz	Type bone scanned	True Positive, n	False Positive, n	False Negative, n	True Negative, n
**Williamson**	Trained	6-view	10	Combined^$^	16	0	0	10
**Hübner**	Trained	4-view	5, 7.5, 8	Radius^#^ /Ulna^#^	58/21^#^	8/4	1/2	18/27
**Chen**	Untrained	4-view	8–12	Separate^+^	48	3	0	17
**Mortiz**	Trained	6-view	9 or 12	Combined	59	2	2	82
**Patel**	Untrained	4-view	7.5	Separate	19	0	0	9
**Ackermann**	Untrained	6-view	7.5	Separate	59	1	2	30
**Weinberg**	Untrained	4-view	7.5–10	Radius/Ulna	10/4^#^	5/1	4/4	21/21
**Chaar-Alvarez**	Trained/Untrained	4-view	10–5	Combined	44*/40^&^	4/15	2/7	51/41
**Sinha**	Untrained	4-view	7–10	Combined	4	0	0	12
**Beltrame**	Trained	6-view	NR	Combined	7	0	1	4
**Eckert**	NR	6-view	10	Separate	61	1	0	53
**Waterbrook**	Untrained	Unclear	12–5	Radius/Ulna	13/3^#^	3/0	1/0	16/23
**Javadzadeh**	Untrained	4-view	NR	Combined	57	4	7	66
**Kozaci**	Untrained	6- view	7.5	Combined	54	1	1	28
**Herren**	Untrained	6-view	7.5	Separate	98	0	0	103
**Musa**	Untrained	NR	NR	Combined	16	0	3	5

^^^ for exact description of training see S2 Text

^/^ for exact description of method of viewing see S2 Text

^$^ combined: distal forearm seen as one entity

^#^ radius and ulna separately, first number is radius, second number is ulna (all the other studies are distal forearm seen as one entity, “separate” are calculated to one entity)

^+^ separate: distal forearm seen as two separate bones

NR: not reported

* Chaar-Alvarez trained

^&^ Chaar-Alvarez untrained.

### Quality of included studies and methodological heterogeneity

The quality of most studies was average to high (Table 3). All studies enrolled patients who would normally undergo conventional x-ray for the diagnosis of distal forearm fracture. Only Moritz *et al*. specifically looked at children with unclear fracture site [36]. Almost all studies were convenience studies, which have a risk of bias in patient selection and none of the studies documented the number of patients who withdrew or didn’t want to join the study. Only one study recorded the number of patients who fell out because of machine malfunction or loss of recorded data [14]. Some of the studies focused as well on ultrasound guided reposition [35,37,41], or axis determination [2,34,37,38,41,42], while most studies excluded patient with clear angulation [2,14,20,31,32,34,36,38–40,43]. Ultrasound and x-ray were consecutively performed in a short period and both imagers were blinded to each other results except for one study, in which this was not clearly described [31]. Ultrasonographers were not blinded to clinical data, which is inherent to performing the examination. There was a different level of training between the ultrasonographers. Some were performed by a (paediatric) radiologist [34,36,39], while others were done by non-trained persons after a short didactic and hands-on training program [2,14,20,32,33,35,37,40–43]. Seven studies used the six-view or comparable method described by Ackerman *et al*. [2,34,36,38,39,41,42], two studies didn’t specify method of viewing clearly [33,43], whilst the others mostly used a 4-view method. Most studies described cut offs for fracture definition by ultrasound, but not all had the same threshold. Probe frequency ranged from 7 to 12 MHz and three didn’t report any probe frequency [39,40,43]. Two studies used one sonographer [35,40], 11 used more than one sonographer (range 2–10 persons) [2,14,20,31–34,36,37,39,41], and four didn’t record how many people performed ultrasound [38,41–43]. All studies except one used conventional x-ray as the reference standard only [32]. Conventional x-rays were reviewed mostly by (paediatric or general) radiologists [2,14,32–39,42], or emergency medicine attending physicians [40,41]. More detailed information about the included studies is described in S2 Text.

**Table 3:** QUADAS-2 risk of bias assessment.

	Risk of Bias				Applicability Concerns		
Study	Patient Selection	Index Test	Reference Standard	Flow and Timing	Patient Selection	Index Test	Reference Standard
**Williamson**	Unclear	Low	Unclear	Low	Low	Low	Low
**Hübner**	Unclear	High	Unclear	Low	Low	High	Low
**Chen**	High	Low	Low	Low	High	Low	Low
**Moritz**	High	Low	Unclear	High	High	Low	Low
**Patel**	Unclear	Low	Low	Low	Low	Low	Low
**Ackermann**	Unclear	Low	Low	Low	Low	Low	Low
**Weinberg**	Unclear	Unclear	Unclear	High	Low	Low	Low
**Chaar-Alvarez**	Unclear	Low	Unclear	Low	Low	Low	Low
**Sinha**	Unclear	Low	Low	Low	Low	Low	Low
**Beltrame**	Unclear	Low	Low	Low	Low	Low	Low
**Eckert**	Low	Low	Low	Low	Low	Low	Low
**Waterbrook**	Unclear	Unclear	Low	Low	Low	Low	Low
**Javadzadeh**	Unclear	Low	Low	Low	Low	Low	Low
**Kozaci**	Unclear	Low	Low	Low	Low	Low	Low
**Herren**	Low	Low	Low	Low	Low	Low	Low
**Musa**	Unclear	Low	Low	Low	Low	Low	Low

### Overall meta-analysis

**Fig 2:**
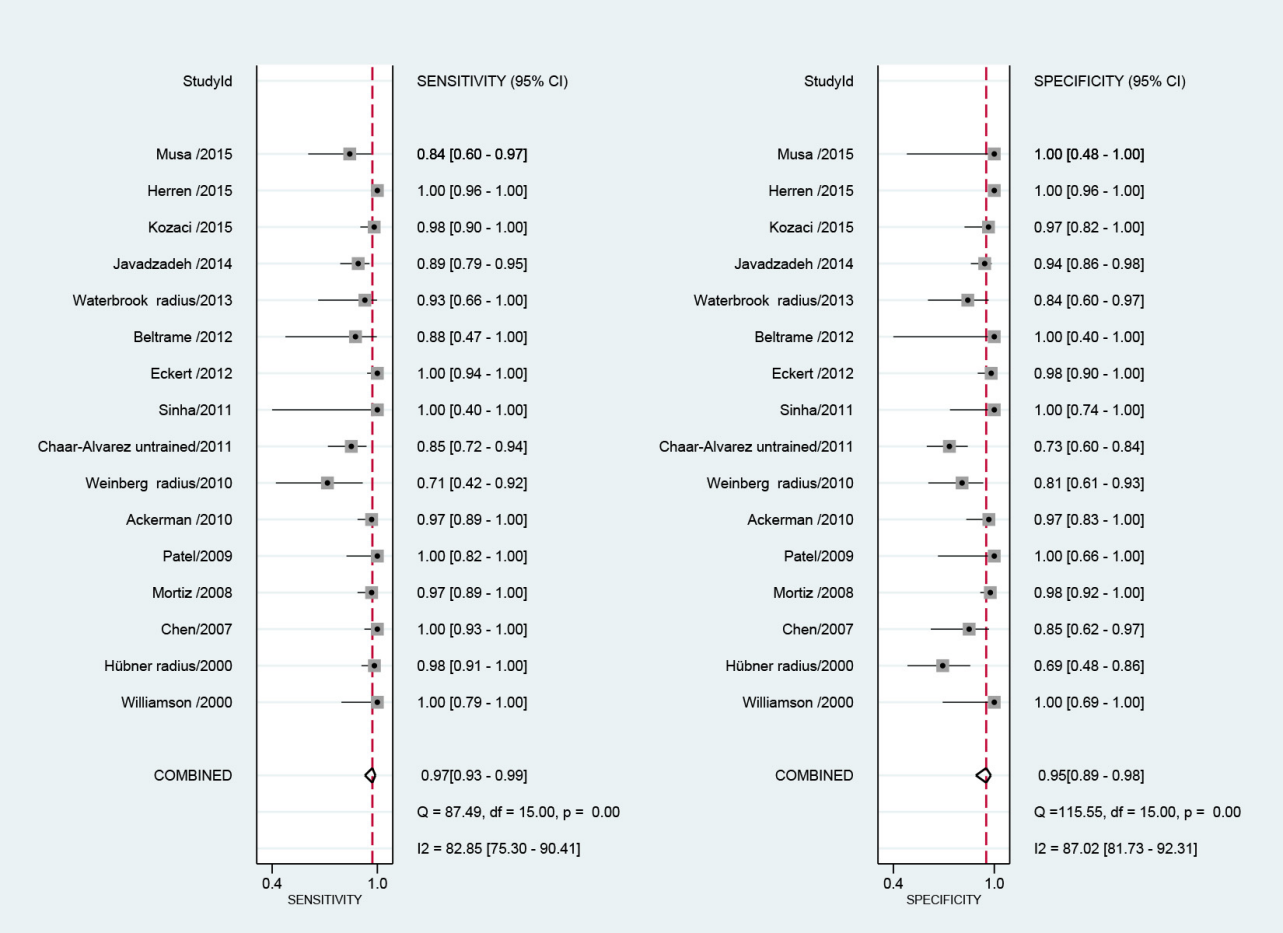
Coupled Forrest plots sensitivity and specificity for ultrasound compared to conventional x-ray, for the diagnosis of distal forearm fracture.

**Fig 3:**
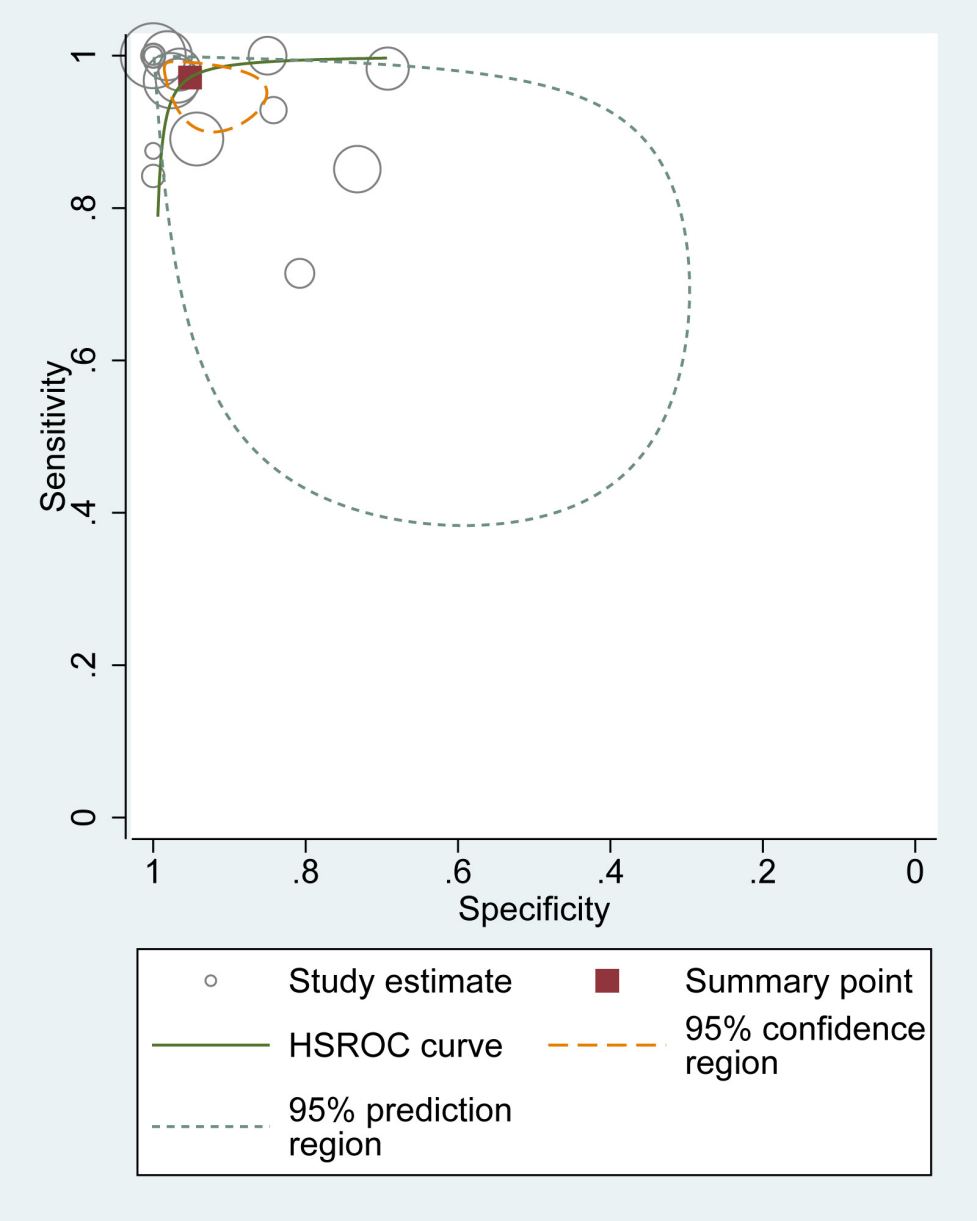
Hierarchical summary receiver operator curve (HSROC) of ultrasound for distal forearm fracture.

Fig 2 shows the coupled forest plots for sensitivity and specificity values of the 16 studies. Pooled estimates of sensitivity and specificity for ultrasound were, respectively, 0.97 (0.93–0.99) and 0.95 (0.89–0.98). The pooled positive and negative likelihood ratio of 20.0 (8.5–47.2) and 0.03 (0.01–0.08), and the corresponding pooled diagnostic odds ratio (DOR) was 667 (142–3,133). The overall HSROC is presented in Fig 3. This reflects high accuracy of ultrasound for the detection of forearm fractures, when compared to the reference standard of conventional x-ray.

### Subgroup analysis by diagnostic imaging

Subgroup analyses are presented in Table 4, for subgroups with 5 or more available studies. No significant difference was detected with respect to probe frequency, skills level, age, or fracture rates. Apparent differences were shown for method of viewing, with the 6-view method showing higher specificity, positive LR, and DOR, than the 4-view method.

**Table 4:** Subgroup analyses.

Variables	Number of studies	Number of participants	Sensitivity	Specificity	+LR	-LR	DOR
**All studies Chaar trained**	16	1,204	0.97 (0.94–0.99)	0.96 (0.91–0.98)	21.9 (10.0–47.9)	0.03 (0.01–0.07)	792 (194–3,236)
**All studies Chaar untrained**	16	1,206	0.97 (0.93–0.99)	0.95 (0.89–0.98)	20.0 (8.5–47.2)	0.03 (0.01–0.08)	667 (142–3,133)
**All studies without radius**	13	1,046	0.98 (0.93–0.99)	0.97 (0.92–0.99)	32.8 (12.0–89.8)	0.03 (0.01–0.07)	1,312 (223–7,703)
**Skills**							
**Trained**	5	369	0.97 (0.93–0.99)	0.94 (0.80–0.99)	16.5 (4.4–62.2)	0.03 (0.02–0.08)	482 (110–2,114)
**Untrained**	11	823	0.96 (0.89–0.99)	0.95 (0.87–0.98)	18.1 (6.8–48.2)	0.04 (0.01–0.12)	448 (68–2,952)
**Not reported**	1	115	-	-	-	-	-
**Method of viewing**							
**4 view**	7	472	0.96 (0.86–0.99)	0.90 (0.81–0.95)	9.83 (4.83–20.00)	0.04 (0.01–0.16)	246 (47–1,297)
**6 view**	7	675	0.98 (0.96–0.99)	0.98 (0.96–0.99)	62.8 (23.2–169.7)	0.02 (0.01–0.04)	4,086 (825–20,232)
**Not reported**	2	57	-	-	-	-	-
**Probe**							
**< = 7.5MHz**	5	490	0.96 (0.89–0.99)	0.95 (0.87–0.98)	18.1 (6.8–48.2)	0.04 (0.01–0.12)	448 (68–2,952)
**>7.5MHz**	8	546	0.97 (0.89–0.99)	0.93 (0.83–0.97)	14.5 (5.4–38.9)	0.03 (0.01–0.13)	503 (49–5,118)
**Not reported**	3	170	-	-	-	-	-
**Age**							
**< = 18 years**	12	951	0.98 (0.93–1.00)	0.96 (0.90–0.99)	26.7 (9.3–76.6)	0.02 (0.00–0.07)	1,490 (171–12,979)
**> 18 years**	3	146	-	-	-	-	-
**Not reported**	2	109	-	-	-	-	-
**Fracture rate**							
**<50%**	7	672	0.94 (0.84–0.98)	0.96 (0.81–0.99)	21.4 (4.3–105.8)	0.06 (0.02–0.19)	370 (26–5,334)
**> = 50%**	9	534	0.98 (0.94–0.99)	0.96 (0.85–0.99)	27.5 (6.1–124.1)	0.02 (0.01–0.06)	1,322 (266–6,573)
**Radius versus ulna**^**#**^							
**Radius**	8	719	0.99 (0.93–1.00)	0.93 (0.83–0.98)	14.8 (5.5–39.8)	0.02 (0.00–0.08)	945 (97–9,201)
**Ulna**	8	669	0.86 (0.75–0.93)	1.00 (0.95–1.00)	254.0 (17.4–3,713.6)	0.14 (0.07–0.26)	1,852 (102–33,781)
**Radius versus ulna**^**##**^							
**Radius**	5	561	0.99 (0.95–1.00)	0.97 (0.91–0.99)	(10.9–112.5)	0.01 (0.00–0.05)	3,860 (440–33,865)
**Ulna**	5	559	0.87 (0.76–0.94)	1.00 (0.96–1.00)	631.6 (21.8–18,277.1)	0.13 (0.07–0.25)	4,8870 (136–174,915)
**Entity**^**#**^							
**Forearm one bone**	6	588	0.99 (0.96–1.00)	0.98 (0.92–0.99)	46.4 (12.0–179.2)	0.01 (0.00–0.05)	5,924 (539–62,967)
**Separate bones**	6	1,176	0.97 (0.94–0.99)	0.99 (0.96–1.00)	73.3 (24.9–215.5)	0.03 (0.02–0.06)	2,394 (582–9,835)

^#^ Subgroups include identical studies

^##^ Subgroup, excluding three studies in which the number of included ulna and radius observations differed

LR likelihood ratio

DOR Diagnostic Odds Ratio.

Eight studies presented separate data on radius and ulna [2,31–33,35,38,41,42]. Comparing these two bones showed a higher sensitivity and LR-, with lower specificity, LR+, and DOR for the radius. Excluding three studies in which the numbers of included ulna and radius observations differed from this analysis did not change this comparison, except that specificity improved for the radius [31–33].

Six studies presented data for the forearm as one entity and data on the separate bones [2,35,37,38,41,42]. Comparing these methods showed no significant differences.

## Discussion

The present meta-analysis demonstrates that ultrasound detects distal forearm fractures in the paediatric age group with a high sensitivity and a high specificity when radiography is used as the reference standard. Ultrasound has an excellent LR + of 20.0 and LR- of 0.03, making it a proficient test to rule in or rule out distal forearm fractures in this young age group. Only three out of hundred patients with a distal forearm fracture will be missed by ultrasound. Furthermore, methods of viewing, and radius compared with ulna were the only sub-analysis which showed significant differences. The 6-view method performed better, with both higher sensitivity (0.98 (CI95% 0.96–0.99)) and specificity (0.98 (CI95% 0.96–0.99)) compared to the 4-view method. The radius had a higher sensitivity and LR-, but lower specificity, LR+, and DOR compared to the ulna. All other sub-analysis including skills level showed no significant differences.

Part of the high accuracy of ultrasound may be explained by the fact that ultrasonographers were not blinded for clinical data. Although ultrasonographers were blinded for the x-ray results, performing ultrasound inevitably is associated with getting clinical information. This might be considered a limitation of the interpretation of this review. We believe however, that this simply reflects daily practice in which the results of ultrasound are always combined with the clinical assessment.

A well-defined protocol for ultrasound in distal forearm fractures should be in place before it can be implemented. The 6-view method views both radius and ulna completely circumferential and reduces the risk of missing (accompanying) fractures. One of the first studies performed described inaccurate ultrasonographic results despite correct detection of a fracture, due to incomplete examination, e.g. examination of a radial fracture without imaging the ulna [31]. Also there is a need for universal definition of a threshold value for fracture as obtained by ultrasound. Up till now some studies used only cortical disruption (e.g. steps, brakes, gap, interruptions) [2,20,32,33,36,39–41], while others also included (subperiostal) hematomas and soft tissue changes [14,31,42]. These different definitions in threshold is probably affecting the different result for fracture detection in Salter Harris 1 fractures in children and subtle fractures [31,32,35,41,45]. In general there should be an uniform technique for visualization and fracture definition in ultrasound for distal forearm fractures.

The measured difference in sensitivity and specificity in our study between ulna and radius is partly explained by the fact that accompanying ulna fractures (especially near the joint) were missed [2,31,35,38,42], and the prevalence of ulna fractures, accompanied or isolated are lower than radial fractures [35,42,44]. This results in a lower sensitivity compared to the radius, but specificity remained high. All isolated ulna fractures [2,35,38,41,42], normally caused by direct impact and thus have a clear point of maximum pain, were detected by ultrasound. The effect of scanning protocol is again visible in the wide CI95% and lower specificity of the subgroup analysis radius/ulna with and without the three studies with unequal visualized radius and ulnas.

Although some authors suggested that sensitivity improves with exposure [32,46], our results supports the idea that ultrasound can be conducted after minimal training for the diagnosis of distal forearm fractures, because there was no significant difference between trained and untrained personnel according to diagnostic accuracy. No sophisticated modules or learning systems are needed before ultrasound can be implemented in clinical practice. Even in the hands of minimally trained personnel ultrasound performed well both as a rule-in and rule-out test for distal forearm fracture. Because distal forearm fractures are common, people will quickly reach the recommended 25 documented and reviewed cases to have received enough competence to perform and interpreted ultrasound on their own [47].

Probe frequency did not affect sensitivity and specificity, and distal forearm seen as one entity or two separate bones can be compared with each other, but caution should be given to studies who have unequal numbers of radius and ulnas scanned, because of incomplete scanning of the distal forearm.

### Strengths and weaknesses

There are several strengths and weaknesses both related to our review technique used, and the obtained data.

We have applied a broad search, using non-restrictive search terms. We did not use x-ray as a separate MeSH heading, Emtree or key word in Embase and Pubmed. This might be considered a limitation. However, since conventional x-ray is the first line diagnostic test, all ultrasound tests were compared to x-ray. We therefore believe that no relevant studies will be missed.

Although gray literature is increasing daily due to Internet, we decided not to include these, as well as research abstract from meeting proceedings or unpublished studies. Both are not commonly subjected to exhaustive peer-review and they provided limited data. This might imply that potential relevant studies were not included.

Another possible limitation may be that we used conventional x-ray as reference standard comparator, which is not the golden standard to detect fractures. Occult fractures occur in convention x-ray in about 2–36% due to overlapping structures, under-mineralized ossification centres and non-perpendicular x-ray beam to the fracture line [18,48]. Because the included studies compared ultrasound to conventional x-ray, true fractures seen on ultrasound, but missed by conventional x-ray were labelled as false positive. Actual sensitivity could even increase further if these fractures were labelled rightly.

Several studies provided impressive diagnostic results with 100% sensitivity and specificity [20,34,37,42]. This may be explained partly by selection bias, small patient groups, and imaging protocol.

A clear strength of this review is the large number of studies performed in children. A weakness is the absence of studies on adults. This might be explained by selection bias and publication bias. We faced insufficient data to perform a reliable sub-analysis for pooled sensitivity and specificity in adults. Only two studies were published that looked at distal forearm fractures in adults alone [39,40], and two in both children and adults [33,41]. One of the first studies performed, described that diagnostic errors occurred mostly nearby areas of bone ends or joints, small bones of hands and feet, non displaced epiphyseal fractures, or in fractures with less than 1-mm fracture line [11,31]. In adults, fractures are more often intra-articular and can have artefacts, due to osteoarthritis, which can lead to misclassification [38]. This is in contrast to children who have a wide range of fractures uniquely seen in children such as torus/greenstick and bending fractures. These fractures have a large cortical component, which makes ultrasound visualization easier. Another reason that more studies focused on the paediatric group may be due to increased radiation sensitivity.

Although most studies excluded patients with clear angulation, studies had a high fracture rate overall (53.2%) and some (especially those focusing on ultrasound guided reposition) also had a high reposition rate (27.3%). Because none of these studies documented the total number of patients visiting the hospitals during the study period with fracture rate of the total group, it is unclear if selection bias has falsely increased fracture rate in some studies, or if there are quite regional differences in fracture prevalence or different practices for ordering x-ray. Fracture rate has been described in literature between 20–50% [49,50].

Another strength of this review is the possibility to perform subgroup analyses, on skills level, methods of scanning, bones scanned (ulna, radius or combined), probe frequency, age, fracture rate, and reposition rate. Our results are in line with a recently published systematic review evaluating ultrasound as a possible alternative to radiographs in diagnosing metaphyseal forearm fractures in children [51]. The sample size in that review didn’t allow definite conclusions about assessing equivalence or non-inferiority of ultrasound compared to x-ray for that indication.

### Implications for daily practice

Implementation of new diagnostic imaging in existing health systems can expect resistance on different levels.

One possible explanation for this resistance is that x-ray has a greater field of view for detecting associated injuries (e.g. scaphoid fracture), which might not be detected with ultrasound examination at a single point of view. However, all imaging techniques should always be interpreted within the clinical context of a patient. In general, it should be noticed that both for x-ray and ultrasound a basic level of training and knowledge is necessary before it can be performed and used accurately in daily clinical practice.

Furthermore, different specialists and medical personnel are involved in the diagnosis and treatment of patients with distal forearm fractures. Conventional x-ray is widely used, stored and easily retrieved by all personnel. Although conventional x-ray of the distal forearm has a very low radiation dose (only 0.16μSv) [50], we should be the children’s advocate and push the system to make it more user-friendly by obtaining the best test with the least risk in each patient [52]. In this respect, ultrasound is preferred over conventional x-ray. An additional benefit of ultrasound is that experienced pain was on average lower in ultrasound compared to conventional x-ray [14,15]. Ultrasound gel itself gives mostly a cooling effect and the patient can keep his/her arm in maximum antalgic position, while the sonographer moves the probe around. With conventional x-ray however, the patient needs to change his arm position to get a proper antero-posterior and lateral x-ray.

Ultrasound has already shown usefulness in the follow up of fracture healing, where callus and fracture union on ultrasound predates its appearances compared to radiographs [53–55]. Also, ultrasound-measured axis compared to conventional x-ray do show comparable results [2,31,37,38,42,56], and ultrasound is increasingly used to determine successful realignment of forearm fractures during closed reductions [57–61].

Although a systematic review by Joshi *et al*. concluded that their evidence does not support replacing conventional radiography with ultrasound for fracture diagnoses [27], we do conclude that, even with all the before mentioned limitations, ultrasound for the diagnosis of distal forearm fractures in children using the 6-view method is a reliable method that equals conventional x-ray in diagnostic accuracy.

Our difference in conclusion is explained by the fact that we only included distal forearm fractures, while Joshi et al included extremity fractures in general. Secondly because of a longer inclusion period we included more studies, with the possibility to perform a meta-analysis showing a high sensitivity and specificity, comparable to conventional radiography. Therefore, it is worth trying to implement ultrasound in existing hospital systems because of the the absence of radiation exposure and reduction in patient discomfort when imaging.

## Conclusion

Ultrasound is a reliable alternative for the diagnosis of distal forearm fractures in the paediatric age group when used by proper viewing method, which has the advantages of being radiation free. To keep quality of existing diagnostic pathways in place we recommend a prospective study using ultrasound from start to end next to conventional x-ray.

## Supporting Information

S1 Text Pubmed and Embase search. (DOC)

S2 Text Other and more specific characteristics of included studies. (DOC)

S3 Text Reason for final exclusion 23 studies. (DOCX)
