# Supplementary material for: Ultrasound for Distal Forearm Fracture: A Systematic Review and Diagnostic Meta-Analysis
Source: PLoS One. 2016 May 19;11(5):e0155659. doi: 10.1371/journal.pone.0155659 (PMC4873261; doi:10.1371/journal.pone.0155659)
Supplement: S1 Text — (DOC) [file pone.0155659.s001.doc]

**S1 Text**

**Pubmed and Embase search**

**Ultrasound for distal forearm fracture: a systematic review and diagnostic meta-analysis**

**Djoke Douma-den Hamer, MD^1^, Marco H. Blanker, MD, PhD^5^, Mireille A. Edens, PhD^2^, Lonneke N. Buijtenweg, MD^1^, Martijn F. Boomsma, MD^3^, Sven H. van Helden, MD, PhD^4^, Gert-Jan Mauritz, MD, PhD^1^.**

**Pubmed search:**

**("Forearm Injuries"[Mesh] OR “forearm injuries”[tiab] OR “forearm injury”[tiab[ OR "Wrist Injuries"[Mesh] OR “wrist injuries”[tiab] OR “wrist injury”[tiab] OR "Ulna Fractures"[Mesh] OR “ulna fracture*”[tiab] OR "Radius Fracture*"[Mesh] OR “radius fracture*”[tiab] OR “wrist fracture*”[tiab] OR “forearm fracture*”[tiab] OR “fore arm fracture*”[tiab] OR “antebrachial fracture*”[tiab] OR “fractura antebrachii”[tiab] OR “distal radius fracture*”[tiab] OR “fractura radii”[tiab] OR “radial fracture*”[tiab] OR “ulnar fracture*”[tiab] OR “ulna distal fracture*”[tiab] OR “distal radius”[tiab] OR (forearm[ot] OR fracture*[ot])**

**AND**

**("Ultrasonography"[Mesh] OR ultrasonography[tiab] OR "ultrasonography" [Subheading] OR "Ultrasonics"[Mesh] OR “ultrasonics”[tiab] OR “ultrasound”[tiab] OR “ultrasonic”[tiab] OR “sonification”[tiab] OR “sonication”[tiab] OR “ultra sound”[tiab] OR “ultrashell”[tiab] OR “ultrasound measurement”[tiab] OR “echography”[tiab] OR “sonography”[tiab] OR “echogram”[tiab] OR “echoscopy”[tiab] OR “echosound”[tiab] OR “sonogram”[tiab] OR “ultrasonic examination”[tiab] OR “ultrasonic diagnosis”[tiab] OR “ultrasonic echo”[tiab] OR “ultrasonic examination”[tiab] OR “ultrasonic scanning”[tiab] OR “ultrasound diagnosis”[tiab] OR “ultrasound scanning”[tiab] OR “ultrasonic detection”[tiab])**

Embase search

'colles fracture'/exp OR 'wrist fracture'/exp OR 'wrist fracture':ab,ti,de OR 'forearm fracture'/exp OR 'forearm fracture':ab,ti,de OR 'fore arm fracture':ab,ti,de OR 'antebrachial fracture':ab,ti,de OR 'fractura antebrachii':ab,ti,de OR 'radius fracture'/exp OR 'radius fracture':ab,ti,de OR ‘radius fractures’:ab,ti,de OR 'distal radius fracture':ab,ti,de OR 'fractura radii':ab,ti,de OR 'radial fracture':ab,ti,de OR 'ulna fracture'/exp OR 'ulna fracture':ab,ti,de OR ‘ulna fractures’:ab,ti,de OR 'ulnar fracture':ab,ti,de OR 'ulna distal fracture':ab,ti,de OR ‘forearm injuries’:ab,ti,de OR ‘forearm injury’:ab,ti,de OR ‘wrist injuries’:ab,ti,de OR ‘wrist injury’:ab,ti,de

AND

‘ultrasound’/exp OR ‘ultrasound’:ab,ti,de OR ‘ultrasonic’:ab,ti,de OR ‘ultrasonics’:ab,ti,de OR ‘sonication’:ab,ti,de OR ‘sonification’:ab,ti,de OR ‘ultra sound’:ab,ti,de OR ‘ultrashell’:ab,ti,de OR ‘ultrasonic measurement’:ab,ti,de OR 'echography'/mj OR ‘echography':ab,ti,de OR ‘sonography’:ab,ti,de OR ‘echogram’:ab,ti,de OR ‘echoscopy’:ab,ti,de OR ‘echosound’:ab,ti,de OR ‘sonogram’:ab,ti,de OR ‘ultrasonic detection’:ab,ti,de OR ‘ultrasonic diagnosis’:ab,ti,de OR ‘ultrasonic echo’:ab,ti,de OR ‘ultrasonic examination’:ab,ti,de OR ‘ultrasonic scanning’:ab,ti,de OR ‘ultrasonography’:ab,ti,de OR ‘ultrasound diagnosis’:ab,ti,de OR ‘ultrasound scanning’:ab,ti,de
