# Supplementary material for: Ultrasound for Distal Forearm Fracture: A Systematic Review and Diagnostic Meta-Analysis
Source: PLoS One. 2016 May 19;11(5):e0155659. doi: 10.1371/journal.pone.0155659 (PMC4873261; doi:10.1371/journal.pone.0155659)
Supplement: S2 Text — (DOC) [file pone.0155659.s002.doc]

**S2 Text. Other and more specific characteristics of included studies**

**Ultrasound for distal forearm fracture: a systematic review and diagnostic meta-analysis**

**Djoke Douma-den Hamer, MD^1^, Marco H. Blanker, MD, PhD^5^, Mireille A. Edens, PhD^2^, Lonneke N. Buijtenweg, MD^1^, Martijn F. Boomsma, MD^3^, Sven H. van Helden, MD, PhD^4^, Gert-Jan Mauritz, MD, PhD^1^.**

**Table 1 Ultrasonographer and other** characteristics of included studies

| **Study** | **Operator** | **Number of Ultrasonographers** | **Training of Ultrasonographers** | **Radiograph interpretation** | **Inter-rater Reliability** | **Method of sonography** | **Probe** | **US Machine** | **Fracture signs** |
| --- | --- | --- | --- | --- | --- | --- | --- | --- | --- |
| **Williamson** | Radiologist | 2 | Consultant radiologist | Formally reported on a later date | Not reported | Musculoskeletal hand/wrist protocol | 10 MHz linear array probe | ATL 2000, Bothell, USA | Steps, brakes, etc |
| **Hübner** | Pediatric surgeons | 3 | Performed > 1500 scans each and attended a training course on scanning bony surfaces | The authors (5) and two additional consultants, compared the ultrasound and radiological findings to confirm the incidence of fractures | Not reported | 4 planes longitudinally, transverse planes in special cases only | 5 MHz and 7.5 linear probe, a water stand-off was added as needed and in later stages an 8 MHz was also used | Sonoline; Siemens/ Nürnberg, Germany | Interruptions, steps or axial deviations on the bone surface and additional findings periosteal lesions, haematomas and sof-tissue changes |
| **Chen** | Emergency Department Physician | 1 | American College of Emergency Physicians-sponsored course and hands-on training in the ED for 1 month | Final readings by attending radiologist | Not reported | 4 views: longitudinal and sagittal views of the radius and ulna from elbow to wrist | 8-12 MHz linear transducer | Sonosite 180, Bothell, Wash | Not reported |
| **Moritz** | Pediatric radiologists | 4 (all in all though 4 pediatric radiologists participated in this study) | Pediatric radiologists | 2 (4) pediatric radiologists | Not reported | All areas were imaged from different directions and in at least two different planes. | 9 or 12 MHz linear transducer | Siemens Sonoline Elegra, Erlangen, Germany | Cortical interruption, cortical step or cortical bulging, or in case of older fracture external callus (irregular bulging of the fractured bone) |
| **Patel** | Pediatric emergeny medicine physicians | 3 | 2 hour didactic and practical session and performed 2 practice BUS examinations supervised by the lead investigator | Attending pediatric radiologists final interpretation | Not reported | Longitudinal plane on the dorsal en lateral aspects of the forearm or leg at each injury site | 7.5 MHz linear small parts probe | Sonoline G40 (Siemens, Mountain View, CA) | Not recorded, only axis measurement 2 intersecting lines along the edge of the cortex |
| **Ackermann** | 5 residents in training, 3 consultants | 8 | Theoretic session in methodic (ablauf, dokumetnation, schallkopf, schnittebenen, achsbestimmung) | Radiologist (independent) | Not reported | Six points (radius longitudinal palmair, radial and dorsal and ulna longitudinal palmair, ulnar and dorsal) | 7.5 MHz linear array transducer | Not reported | Cortical gap, a kink, a torus formation, or a displacement |
| **Weinberg** | Pediatric emergency physicians | 10 | 1 h of point-of-care musculoskeletal training, consisting of a 30 min lecture viewing ultrasound video of various fractures, followed by a 30-min practical hands-on scanning session of normal bone anatomy and simulated fractures | Attending radiologists reading | Not reported | Perpendicular orthogonal planes (long and short axis), additional oblique or longitudinal views as necessary | 7.5-10 MHz linear array transducer | Sonosite Micromaxx, Bothell, WA and Siemens GS60, Mountain View, CA | Cortical interruption or irregularity, elevation of posterior fat pad |
| **Chaar-Alvarez** | Pediatric Emergency Medicine Physicians (2) or fellows (2) | 4 | Completed training in emergency ultrasonography through a certified bedside emergency US course. | Official dictated reading from a board-certified radiologist  (inconsistentie blinded US/Xray pediatric radiologist independ) | 0.57 (0.41-0.73)  McNemar 1.14 | 4 views (longitudinally distal radius/ulna in coronal and anterioposterior planes). Additional views were obtained if they were felt to be helpful | 10-5 MHz broadband linear array transducer | SonoSite Titan (SonoSite, Bothell, Wash) | Subperiosteal heamtomas, beding or plastic deformity, cortical disruption, and reverberating echoes |
| **Sinha** | EP’s: consultant in emergency medicine, two senior resident in orthopedic, one senior resident in surgery | 4 | 1-day didactic program followed by a hands-on training session, 10 positive and 10 negatives supervised scans | Blinded orhopedic specialist | Not reported | Transverse and longitudinally plane | 7-10 MHz linear array | Not reported | Skip or any defect in the cortex |
| **Beltrame** | Radiologist | 3 | Radiologist | Radiologist (3) | Not reported | Longitudinal plane on the dorsal/ventral en lateral aspects | Not reported, with or without gel pad | Siemens Elegra and Sonosite Titan | Cortical disruption, visible as a step-off or as an interruption in an otherwise continuous line |
| **Eckert** | Not reported | Not reported | Not reported | Independent radiologist | Not reported | 6 planes dorsal/palmar/ ulnar/radial radius en ulna | 10 MHz linear array | Not reported | Not reported |
| **Waterbrook** | 2 atteending physicians, 3 sport medicine fellows, 3 residents, and a fourth-year medical student | 9 | 2 attending physicians had prior training in using POCUS (FAST)  Fellows, residents (both also active POCUS training programme) and medical students received a 15 minute in-service from the principal investigator on diagnosis of long bone fracture using ultrasound | Final radiology interpretation by a radiology attending physician | 96.4% with a k of 0.921 (95% CI: 0.612-0.986) | Transverse and longitudinal views for all areas examined | 12-5 MHz broadband linear aray | Zonare (Zonare Medical Systemss, Montain View, CA), Ultrasonix (Ultrasonix Medical Corporation, Richmond, British Columbia, Canada), or Sonosite (Sonosite, Borthell, WA) | Break, step-off, or irregularity in the bony cortex |
| **Javadzadeh** | 2^nd^ year emergency medicine resident | 1 | Hands on training, 12 positive and 12 negative scans before start of study | EM attending physician | Not reported | Two perpendicular planes | With and without waterbath  Linear probe | Not reported | A breach in the continuity in the cortex |
| **Kozaci** | Emergency physicians | Not reported > 2 | 30 minutes didactic and 30 minute practical standard POCUS training | Emergency physicians  (1 hour direct radiography training for evaluation of DRFs) | Not reported | Longitudinal and lateral images of distal radius from anterior and posterior in longitudinal and transverse planes | 7.5 MHz linear transducer | Esaote, Firenze, Italy | Cortical disruption, (type fissure, linear, fragmented, torus) Angulation and step off were also measured |
| **Herren** | Residents 2^nd^ to 6^th^ year traumatology | Not reported | 30-minutes training, and reference manual | Attending experts in radiography | Not reported | 6 planes: dorsoradial, radial, palmar radial, palmar ulnar, ulnar and dorsoulnar | 7.5 MHz linear transducer | Siemens AG, Cologne, Germany | Also axis deviation, cortical gap, cortical bulging, cortical deviation or a positive hematoma covering the corticalis in all views |
| **Musa** | ENPs and doctors | Not reported | Three two hour training sessions by manufacturer of ultrasound plus training session before study | Several episodes of practice involving patients with normal and abnormal Xray | Not reported | Not reported | Not reported | Not reported | Not reported |

**Tabel 2: inclusion/exclusion criteria, pain/discomfort, duration and some comments**

| **Study** | **Inclusion** | **Exclusion** | **Pain/discomfort** | **Duration** | **Comments** |
| --- | --- | --- | --- | --- | --- |
| **Williamson** | Children 2-14 years  Suspected isolated, undisplaced, non-articular forearm fracture | Clinical evidence of deformity | No reports of discomfort | Not reported | In future validate angulation and 1 hour training |
| **Hübner** | Children  Suspected fracture | Open fractures class 2 and 3, obviously deformity or unstable fractures | In most cases without distress to the children | 5-10 minutes | 11.6% inaccurate results due to incomplete examinations, e.g. examination of a radial fracture without imagining of the ulna, quality improved with experience.  It was less dependable for compound injuries, fractures adjacent to joints, lesions of the small bones of the hand and foot, non-displaced epiphyseal fractures or those with a fracture line of less than 1 mm |
| **Chen** | Patients 2-21 years  Suspected forearm fracture  When investigator available | Open fracture, evidence of neurovascular compromise, associated elbow injury | No significant pressure was needed to visualize the bone, no additional pain medications were needed | Less than 2 minutes | Ultrasound guided reduction (26 of 48fractures)  Might be additional evidence of subtle physeal injuries (SH1) with small amounts of internal bleeding |
| **Moritz** | newborn to 17 years old nonspecific clinical signs or clinically indistinct fracture location  all patients had an ultrasound as well as an X-ray examination of the traumatized part of the body | In cases of positive radiographs, the examination was finished without ultrasound to avoid unnecessary, probably painful examination  Patients with typical symptoms of a fracture and only X-ray examination were not included in this study. | The pain was centered at the site of the fracture when the probe was in contact. Although the increased pain caused more restlessness especially in infants, the overall result was easier indentification of the fracture and not additional difficulty with the examination | In cases of evident fracture signs the examination time was up to 5 minutes. Cases of minimal fracture signs required very thorough examinations lasting up to 15 minutes. | All patients underwent clinical follow-up within one week  Especially in children younger than 6, the detection of subtle details is very difficult due to patient restlessness.  Ultrasound sens 92.2%, specificity 99.5%, X-ray sens 93.2%, spec 99.8% (unclear fracture localization) |
| **Patel** | 2 through 17 years with suspected radius, ulna, tibia or fibula fracture  When one of participating physicians was available | Open fractures, neurovascular compromise, hemodynamic instability, suspected fractures likely involving joints | BUS examination did not exacerbate pain | Usually less than 5 minutes | Degree of angulation and the distance of displacement were also determined by ultrasound  2 false positive turned to be real fractures by follow up X-rays |
| **Ackermann** | 0-12 years with suspected closed forearm fracture, defined by a mechanism of injury consistent with forearm injury, bony tenderness, and absence of open wounds | Open wounds, a deformity of more than 30 degree, and lesions of neural or vascular structures requiring immediate operation | Ultrasound-gel often having a cooling and pain-reducing effect | Not reported | Difference between the means of deformities were 1.6, 1.6, 0.2 and 0.2 degree  Four oblique views can be added for the ulnar edge of the radius and radial edge of the ulna |
| **Weinberg** | Patiens < 25 years with musculoskeletal injury requiring X-ray or CT, the injured body contained a bone with an identifiable linear cortex on point-of-care ultrasound; eligible long bones included the humerus, radius, ulna, femur, tibia and fibula, eligible non-long bones included skull, mandible, clavicle, rib, metacarpal, phlanx, patella and metatarsal | Gross deformity, arrival with prior diagnosis of fracture on X-ray, hemodynamic instability, need for emergent surgery, laceration over the injury or suspicion open fracture | 0 patients reported discomfort/pain during POC ultrasound | Median time 4 minutes | End-of-bones and near joints most errors happened due to curved sometimes irregular contours (>85%)  Missed fracture involving paired adjacent bones that were not examined by ultrasound were also included in the analysis of test performance characteristics  Overall relative high specificity, relative low sensitivity |
| **Chaar-Alvarez** | 1-17 years, nonangulated distal forearm injuries, history of recent trauma to the forearm and a normal neurovascular examination distal to the injury | Clinical forearm deformity, multisystem trauma, altered metal state, hemodynamic instability, previous radiography performed at an outside institution, developmental delay, open wounds to the forearm, allergy to US gel, or extremity pain/swelling at sites proximal or distal to the injured forearm that would necessitate radiographs | Mean FACES pain scores were higher following radiography than US (1.7 vs 1.2, p = 0.004) | Usually take less than 5 minutes | Over diagnosis of fracture by bedside ultrasonographers  Learning curve |
| **Sinha** | Pediatric patients up to the age of 17 years with complaints of post-traumatic arm, elbow, wrist, leg, and ankle pain | Sustained injury >72 hours prior to presentation, previous fracture, obvious deformity, femur fractures, spine or pelvis injuries and life- and limb-threatening injuries | Not reported | Not reported | One fracture missed at elbow, near joint |
| **Beltrame** | Positive clinical history of trauma and suspected fracture on objective clinical examination (long bones, ribs/stenum/clavicle, nasal bones or phalanges) | Hemodynamic instability, open fractures, neurovascular lesions, suspected fractures probably involving joints, deformities indicative of fractures (e.g., angulations) and traumatized bones containing orthopedic hardware | Generally well tolerated | Never took more than 5 minutes | US of bone injuries not involving joints may be comparable to radiography for identifying fracture (hands/feet 75% agreement) |
| **Eckert** | Clinical and history forearm fracture | Open fracture, clear deformity, neurovascular lesions | Gel well accepted (cooling effect) | On average 2-3minutes | 56 patient axis determined, 1.7 degree difference |
| **Waterbrook** | Complaints of long bone trauma, medically stable, wer not altered, had symptoms of a possible long bone fracture at any location along the bone in question, and required radiographic imaging  Investigator available | Medically unstable, open fractures | No patient complained of discomfort and in fact anecdotally noted in a few cases that the ultrasound examination was more comfortable than the radiographic examination | Not reported | Sensitivity for intra-articular fracture 90.9%  Sensitivity lower, prevalence only 29%  Specificity high, rule out fracture, pretest and post test probability  All ages |
| **Javadzadeh** | History of hand, wrist, or distal forearm trauma not more than 72 h prior to admission | Open fractures, obvious deformity, lacerations, previous fracture on the same site | Not reported | Not reported | Water bath 12.5 statistically different with and without  Adults only |
| **Kozaci** | 5-55 years with simple low-energy extremity trauma | Prediagnosed radius injury, open fractures, neurovascular injury, fracture with dislocation, other systemic injuries, unstable vital signs, or life-threatening injury | Not reported | Not reported | Treatment decision  Age 5-55  23 (55fractures) deformity on physical examination  No problems near joint space/SH 1 |
| **Herren** | Pain in the forearm following an adequate trauma | Open wounds, peripheral disorders of sensitivity and/or circulation, axis deviation that required immediate reduction or pre-existing deformities of the forearm, age limit 11 years | Agreeable cooling effect of gel | Not reported | Also axis deviation |
| **Musa** | High clinical suspicion of closed fracture | Injuries other than distal limb, below age of 2, bleeding or laceration near fracture side and clinical deformity | Less painful and distressing than xray |  | Can rule in fracture, but not rule out |
