# Supplementary material for: Ultrasound for Distal Forearm Fracture: A Systematic Review and Diagnostic Meta-Analysis
Source: PLoS One. 2016 May 19;11(5):e0155659. doi: 10.1371/journal.pone.0155659 (PMC4873261; doi:10.1371/journal.pone.0155659)
Supplement: S3 Text — (DOCX) [file pone.0155659.s003.docx]

**S3 Text**

**Pubmed and Embase search**

**Ultrasound for distal forearm fracture: a systematic review and diagnostic meta-analysis**

**Djoke Douma-den Hamer, MD^1^, Marco H. Blanker, MD, PhD^5^, Mireille A. Edens, PhD^2^, Lonneke N. Buijtenweg, MD^1^, Martijn F. Boomsma, MD^3^, Sven H. van Helden, MD, PhD^4^, Gert-Jan Mauritz, MD, PhD^1^.**

| Excluded articles | Reason for exclusion |
| --- | --- |
| Ackerman 2013 | Abstract |
| Ackerman 2009 | Shared identical population as with 2010 |
| Ang 2010 | Study design did not fulfill inclusion criteria: focused on fracture reduction |
| Anast 1958 | Not relevant: article about velocity, pre imaging area |
| Barata 2012 | Study design did not fulfill inclusion criteria: unable to construct 2x2 tables |
| Bischoff 2013 | Review: commentary |
| Chaar 2009 | Abstract |
| Cohen 2000 | Study design did not fulfill inclusion criteria: follow up confirmed fractures |
| Cross 2011 | Review article |
| Dallaudiere 2015 | Study design did not fulfill inclusion criteria: upper extremity, no specification between type of upper extremity fractures |
| Dubrovsky 2015 | Study design did not fulfill inclusion criteria: focused on fracture reduction |
| Eckert 2014 | Review article on the use of US in pediatric fractures |
| Eckert 2012 | Shared identical population as 2012 but smaller group 76 instead of 115 patients |
| Giardino 2002 | Not relevant: osteoporosis |
| Hollevoet 2000 | Not relevant: osteoporosis |
| Karabay 2013 | Review: more general wrist problems |
| Kirchpatrick 2003 | Case report |
| Mack 2006 | Abstract |
| Marshburn 2004 | Study design did not fulfill inclusion criteria: humerus and femoral fractures only |
| Pountos 2010 | Study design did not fulfill inclusion criteria: either x-ray or ultrasound not and |
| Rathfelder 1995 | Study design did not fulfill inclusion criteria: 31 children only ultrasound, not x-ray |
| Sapozhnikov 1987 | Russian article |
| Simanovsky 2009 | Study design did not fulfill inclusion criteria: only performed if no fracture on x-ray |
